# Comparative short‐term efficacy of endoscopic sinus surgery and biological therapies in chronic rhinosinusitis with nasal polyps: A network meta‐analysis

**DOI:** 10.1002/clt2.12269

**Published:** 2023-06-01

**Authors:** Jiani Chen, Huan Wang, Chen Zhang, Le Shi, Qianqian Zhang, Xiaole Song, Dehui Wang, Li Hu, Hongmeng Yu, Xicai Sun

**Affiliations:** ^1^ ENT Institute and Department of Otorhinolaryngology Eye & ENT Hospital Fudan University Shanghai China; ^2^ Research Units of New Technologies of Endoscopic Surgery in Skull Base Tumor (2018RU003) Chinese Academy of Medical Sciences Beijing China; ^3^ High Altitude Rhinology Research Center of Eye & ENT Hospital of Fudan University and People's Hospital of Shigatse City Shigatse China; ^4^ Department of Otolaryngology People's Hospital of Shigatse City Shigatse China

**Keywords:** biologics, chronic rhinosinusitis with nasal polyps, endoscopic sinus surgery, quality of life, systematic review

## Abstract

**Background:**

To compare the safety and efficacy between endoscopic sinus surgery and different biologics in treating chronic rhinosinusitis with nasal polyps in adults by reviewing the existing clinical trials.

**Methods:**

Data extraction and risk of bias assessment were conducted by 2 independent reviewers according to the PRISMA recommendations and any disagreement was resolved by a third investigator. Outcomes were measured through a random‐effects model. We searched Embase, Web of Science, MEDLINE, Cochrane, and other relevant sources from its inception to April 30, 2022. We included randomized controlled trials(RCTs) involving endoscopic sinus surgery (ESS) or biologics in treating adult patients with chronic rhinosinusitis with nasal polyps. Studies involving other miscellaneous diseases, non‐RCT design, and insufficient participants or follow‐up were excluded.

**Results:**

In this systematic review, five RCTs and 1748 patients were included. All the biologics, as well as ESS, could significantly improve key nasal outcomes in CRSwNP both at 6 months and 1 year. Dupilumab exhibited better efficacy than ESS in improving SNOT‐22 scores at one year. However, ESS showed superiority over three biologics in improving nasal congestion scores (NCS) at two various time points, except for better efficacy of Dupilumab at 1 year. For the loss of smell scores, a greater improvement was observed in the Dupilumab cohort compared with other biologics and even ESS counterparts. Safety analysis showed no significant difference between the ESS cohort and biologic treatment.

**Conclusions:**

In summary, ESS showed comparable improvement in quality of life and symptoms to Omalizumab, Mepolizumab, and Benralizumab. Dupilumab seems to be more effective than ESS in selected items, whereas head‐to‐head trials and real‐world studies are urgent to compare their efficacy. Our findings also showed that biologics could be applied as alternative or adjuvant therapy for uncontrolled severe CRSwNP.

## 1 INTRODUCTION

Chronic rhinosinusitis with nasal polyps (CRSwNP) is a sinonasal inflammatory disease that affects around 2%–4% of the general population.^1^ CRSwNP is characterized by bilateral nasal polyps and persistent symptoms including nasal congestion, rhinorrhea, loss of smell, and headache/facial pressure, which poses significant impairment of quality of life (QoL) as well as increased economic burden.

The current treatment strategy for CRSwNP aims to reduce local inflammation and achieve disease control. Primary treatment for CRSwNP includes medical therapy, such as topical intranasal corticosteroids, nasal irrigation, antibiotics, and short courses of oral corticosteroids. Endoscopic sinus surgery (ESS) plus medical therapy is always reserved for patients who are unresponsive to appropriate medical therapy.^2^ Numerous studies have demonstrated that ESS plus medical therapy significantly improves symptoms and health‐related Qol in patients with CRSwNP.^3^, ^4^, ^5^ However, long‐term follow‐up data suggest that a high proportion of CRSwNP patients are subjected to disease recurrence after ESS.^6^ Therefore, novel therapy is urgently required for CRSwNP to achieve long‐term control.

Growing evidence has confirmed that the majority of CRSwNP patients in Western populations are characterized by a type 2 immune response, sometimes associated with comorbid asthma. In recent years, emerging research has highlighted a significant role for biological therapies such as human monoclonal antibody drugs targeting type 2 inflammation, including Omalizumab (anti‐IgE), Mepolizumab (anti–IL‐5), Dupilumab (anti–IL‐4 receptor a), Benralizumab(anti‐IL‐5 receptor a), Tezepelumab(anti–TSLP), Lebrikizumab(anti–IL−13), and so on.^7^ Multiple randomized controlled trials (RCTs) showed promising efficacy in reducing polyp size and improving symptoms as well as QoL.^8^, ^9^, ^10^, ^11^ Very recently, a multicenter RCT demonstrated a significant effect of endoscopic sinus surgery with medical therapy over medical therapy alone in terms of main symptoms, QoL, and a reduction in systematic corticosteroid use.^12^ In the new era of biological therapy, the comparison of efficacy between biologics and ESS could guide the treatment choice for both physicians and patients. To date, there are no head‐to‐head RCTs between biological treatments and ESS for CRSwNP. In the absence of such trials with direct comparison, an alternative approach by indirect treatment comparisons (ITCs) can also provide useful information on the relative treatment effect on severe CRSwNP.

The purpose of this study was to conduct an ITC using data from RCTs to compare the efficacy and safety between biologics and endoscopic sinus surgery for patients with severe, inadequately controlled CRSwNP.

## 2 METHODS

### 2.1 Data source and search methodology

We performed a systematic literature review of relevant RCTs comparing the efficacy and safety of endoscopic sinus surgery versus biologics for the treatment of CRSwNP. Search strategies were designed using a combination of medical subject headings (Emtree in Embase, and Medical Subject Headings in Web of Science, MEDLINE, and Cochrane) and free‐text terms to identify relevant RCTs that investigated ESS and biological treatments in CRSwNP from January 1, 1980, to April 30, 2022, without language limitations.

### 2.2 Eligible criteria and selection of studies

Inclusion and exclusion criteria as followed in this meta‐analysis were specified before literature screening.

#### 2.2.1 Inclusion criteria

Articles meeting the following criteria were selected: (1) Population: Adult patients (≥18 years old) visiting the outpatient clinic for CRSwNP with ongoing symptoms (Nasal blockage/obstruction/congestion, nasal discharge, facial pain/pressure, or loss of smell) regardless of standard steroid treatment, nasal rinse, or surgery. (2) Study design: RCTs. (3) Intervention: Endoscopic sinus surgery or all types of biologics to treat CRSwNP. (4) Comparison: Placebo, standard of care, or no treatment.

#### 2.2.2 Exclusion criteria

(1) Studies in which patients have undergone diseases that may influence the outcome results including but not limited to active upper or lower respiratory tract infection, nasal cavity tumors, allergic fungal sinusitis, cystic fibrosis and granulomatosis with polyangiitis. However, asthmatics can be included in the analysis. (2) Non‐RCTs, meeting abstracts, review articles, case studies, case reports, and animal or in vitro studies. (3) Less than 10 participants per treatment arm. (4) Endpoint evaluation earlier than 48 weeks.

Two researchers independently executed the comprehensive study selection process and reached a final consensus on disagreements.

### 2.3 Data extraction and outcome measurements

Data extraction in different trials meeting the criteria above was completed by 2 investigators and any disagreement in this process was resolved by a third investigator. Furthermore, the risk of bias was assessed by 2 independent reviewers according to the PRISMA recommendations.

We used the 22‐item sinonasal outcome test (SNOT‐22) score (ranging from 0 to 110) as our primary outcome. It is a multi‐domain questionnaire, which could evaluate disease‐specific health‐related quality of life in patients with CRSwNP about their rhinological, ear, and facial symptoms, or even long‐term emotional and social influence. Other secondary outcomes available for comparison across the trials were mean change from baseline at 6 months and 1 year in EQ‐5D‐5 L vas score (ranging from 0 to 100), loss of smell score, nasal obstruction score, peak nasal inspiratory flow (PNIF), exacerbation of CRSwNP warranting additional surgery and serious adverse events (SAEs). We analyzed the data for conditions requiring remedial surgery and SAE only at the end of follow‐up (EOF, longer than 48 weeks). For these data, lower scores indicate better outcomes excluding PNIF and EQ‐5D‐5 L vas scores, which present the opposite.

For continuous outcomes, the mean values with standard deviations (SDs) and the number of participants in each arm were collected. The effect of different treatments was calculated on the basis of their changes from baseline, namely absolute differences of the means between the baseline and the post‐intervention measurement. Data were aggregated in the study of multi‐arms and different dosing regimens.

### 2.4 Statistical analysis

Concerning indirect treatment comparison methodology, we conducted Bucher ITC proposed in 1997^13^ for each outcome of interest. Firstly, pooled effects were generated via meta‐analysis from the results of the same biological treatment for the intervention arm versus the control arm. Next, the pooled effects produced in the first step were indirectly compared through a common comparator. The INCS arm served as the common comparator in this meta‐analysis because all these RCTs in CRSwNP compared the efficacy of monoclonal antibodies or endoscopic sinus surgery with an INCS control, although the lack of standardized doses of INCS in the ESS cohort limited the explanation of the efficacy differences in this mata‐analysis. Assuming a random distribution of the real therapeutic effects around the population mean effect, the frequentist model was used to provide conservative estimates by acknowledging the potential heterogeneity in RCTs assessing the same treatment owing to the disparate study design or clinical characteristics of enrolled patients. ITCs were implemented through the network package in Stata SE 15.1 (Stata Corp LLC). We performed quality assessment through Review Manager (version 5.4; The Cochrane Collaboration).

The ultimate goal of this study was to quantitatively analyze the main outcome results. For continuous data, including SNOT22, EQ‐5D‐5 L vas score, loss of smell score, nasal obstruction score, and peak nasal inspiratory flow, we described treatment effects of various intervention methods as mean differences (MDs) along with a 95% CI when outcomes reported in studies were on the same scale. And the standardized mean differences (SMDs) were used for cases of inconsistency in units or methods of measurement. For all dichotomous outcomes, including exacerbation of CRSwNP warranting remedial surgery and serious adverse events, odd ratios (ORs) together with 95% confidence interval (CI) were calculated. Differences were considered significant when *p* ≤ 0.05. Pooled estimates of the mean differences in SNOT22, ED‐5D‐5 L VAS score, and symptom score between different arms were measured through a random‐effects model.

## 3 RESULTS

### 3.1 Literature search and feasibility assessment

**FIGURE 1:**
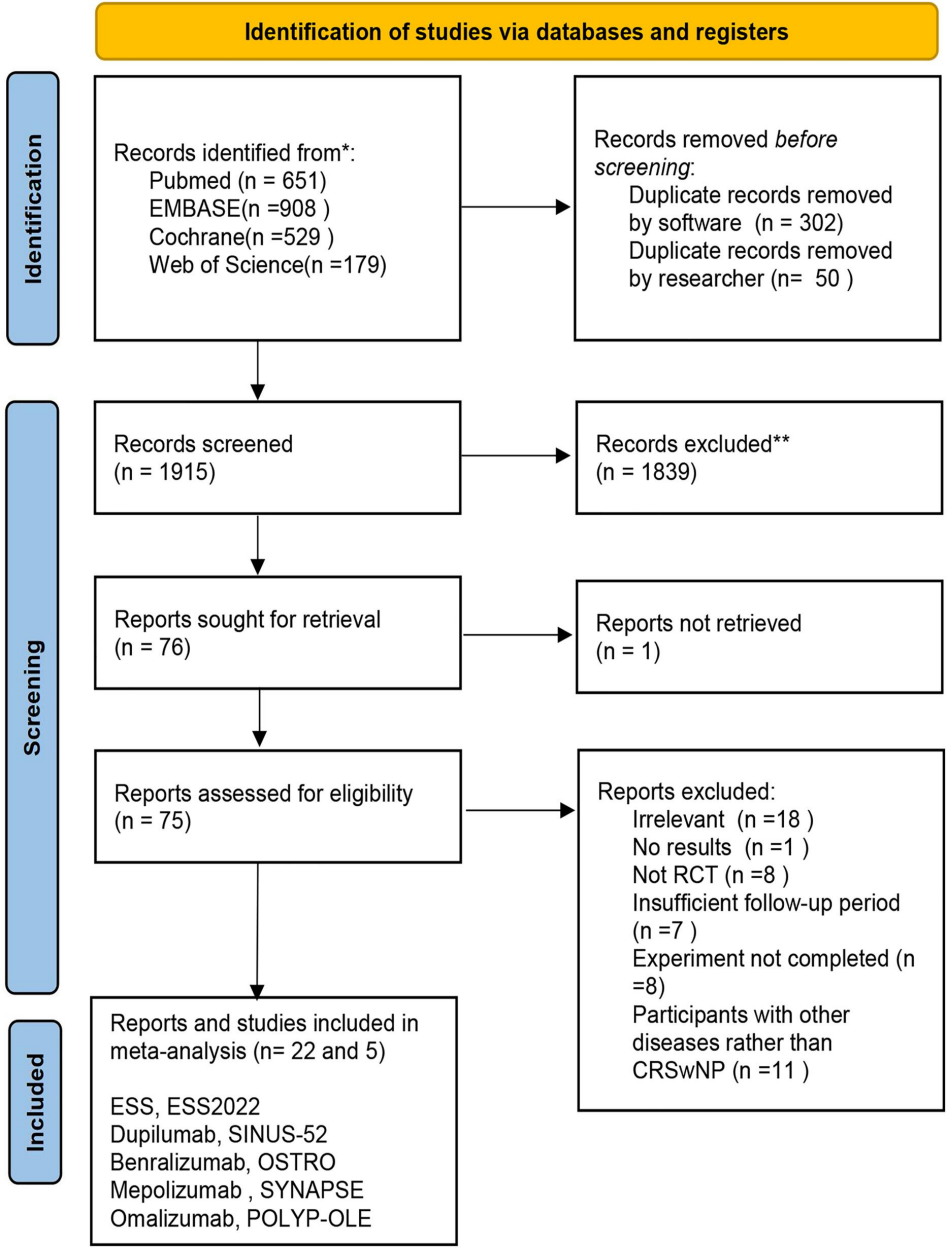
Publication screening strategy. The literature search was conducted specifically for ITC analysis to identify all clinical evidence of ESS, biologics, and conventional treatment used in CRSwNP. CRSwNP, chronic rhinosinusitis with nasal polyps; ESS, endoscopic sinus surgery; ITC, the indirect treatment comparison; RCT, randomized controlled trial.

The systematic initial literature search in various databases identified 2267 records, of which 352 were abandoned after the removal of duplicates by hand and software. After title and abstract screening, there were 75 records available for the full‐text appraisal that met the eligibility criteria. After that, 53 records were retained due to a lack of quantifiable results (*n* = 1), insufficient follow‐up period (*n* = 7), uncompleted experiment (*n* = 8), and so on. Finally, there were 22 records representing 5 unique RCTs eligible for ITC analysis, including ESS2022 [NTR4978], SINUS‐52 [NCT02898454]), OSTRO [NCT03401229], SYNAPSE [NCT03085797] and POLYP‐OLE [NCT03478930] (shown in Figure 1). These 5 RCTs conducted in 2015–2020 could study the efficacy of endoscopic sinus surgery plus medical therapy and four monoclonal antibodies including Dupilumab, Benralizumab, Mepolizumab, and Omalizumab on the treatment of patients with CRSwNP.

We analyzed the schemes of the treatment and control groups and other characteristics in these trials (shown in Table 1). A total of 1748 patients were investigated in the 5 RCTs spanning from 48 to 52 weeks, with 118 of them receiving endoscopic sinus surgery, 832 monoclonal antibodies, and 798 placebos. The protocol of treatment groups is shown in Table 1 and patients assigned to control groups were prescribed placebo with background intranasal corticosteroid (INCS) in biologic trials and any suitable medical therapy except for biologicals in ESS trial. Only one of the RCTs was a multi‐centric study performed in the same country—the Netherlands,^12^ while the other 4 RCTs were all conducted in different countries.^8^, ^9^, ^11^

**TABLE 1:** Characteristics of RCTs included.

Study	ESS2022	OSTRO	SINUS52	SYNAPSE	POLYP‐0LE
Year	2015–2019	2018–2020	2016–2018	2017–2019	2018–2020
Scope	Multicenter	Multicenter	Multicenter	Multicenter	Multicenter
Inclusion criteria	Bilateral CRSwNP; age >17 years; indication for ESS (primary or revision) as judged by their ENT surgeon	Bilateral CRSwNP, NPS ≥5 with unilateral scores≥2, symptoms despite maintenance INCS, history of SCS use and/or surgery for nasal polyps, moderate to severe nasal blockage, SNOT‐22 ≥ 30	Bilateral CRSwNP and symptoms despite INCS; receiving SCS past 2 years OR previous sinonasal surgery; NPS≥5	Nasal obstruction VAS >5; overall symptoms VAS >7; NPS ≥5; history of nasal surgery past 10 years; symptoms despite maintenance INCS	Bilateral CRSwNP; nasal congestion; impaired HRQoL; weight (30–150 kg); serum IgE (30–1500 IU/ml) AND ≥4 weeks of INCS before screening visit 1; total NPS ≥5; NCS ≥2; SNOT‐22 score ≥20
Design	RCT	RCT	RCT	RCT	RCT
Patients enrolled, total TRG, CRG (n)	234; 118; 116	413; 207; 203	448; 145 + 150; 153	407; 206; 201	249; 124; 125
Treatment	Endoscopic sinus surgery	Benralizumab (anti‐IL‐4Rα)	Dupilumab (anti–IL‐4RaMAb)	Mepolizumab (anti‐IL‐5)	Omalizumab (anti‐IgE)
Intervention protocol	Either ESS plus medical therapy within 6 weeks after the baseline visit; or receiving any suitable medical therapy, except for biologics	Either Benralizumab Q4W for the first 3 doses and Q8W thereafter; placebo administered Q4W for the first 3 doses and Q8W thereafter	Either dupilumab subcutaneously 300 mg Q2W for 52 weeks; dupilumab Q2W for 24 weeks, then Q4W thereafter; or placebo Q2W for 52 weeks plus MFNS 100 μg in each nostril twice daily	Either up to 13 SC doses of Mepolizumab 100 mg/mL Q4W to week 52; or a matching placebo Q4W to week 52	Either Omalizumab Q2W/Q4W for 52 weeks; or placebo for 24 weeks then Omalizumab Q2W/Q4W at week 24 at the same dosing schedule
Duration (weeks)	52	48	52	52	52

Abbreviations: CRG, control group; CRSwNP, Chronic rhinosinusitis with nasal polyps; ENT, ears, nose and throat; ESS, endoscopic sinus surgery; HRQoL, Health‐related Quality of Life; IgE, immunoglobulin E; INCS, Intranasal steroids; MFNS, Mometasone furoate nasal spray; NCS, Nasal Congestion Score; NPS, nasal polyps score; Q2W, every 2 weeks; Q4W, every 4 weeks; RCT, randomized controlled trials; SC, subcutaneous; SCS, systemic corticosteroids; SNOT‐22, the 22‐item Sino‐Nasal Outcome Test; TRG, treatment group; VAS, Visual Analogue Scale.

After the qualitative assessment of baseline characteristics for cardinal outcomes of interest (age, BMI, nasal polyp score(NPS), Lund‐Mackay CT score, the percentage of comorbid asthma, etc.), we found that data were mostly comparable among these included randomized controlled trials at baseline (shown in Table 2). In terms of main baseline indicators like the percentage of comorbid asthma, SNOT‐22, or objective characteristics like LMS and serum total IgE, it seems that the disease severity in the ESS trial is no lower than that in the Dupilumab study (shown in Table 2). However, the baseline percentage of previous nasal surgery in the SYNAPSE trial was much higher due to the specific inclusion criterion which recruited patients who had at least one previous surgery in the past 10 years. All participants had persistent bilateral nasal polyps and varying degrees of nasal symptoms. Patients in monoclonal antibody groups were required to have an NPS >5(8–11), whereas those in the ESS group were required to have indications for endoscopic sinus surgery judged by ENT surgeons.^12^ Since the conduction of the ESS study was not as rigorous as FDA trials just like those biologics studies in our analysis, the researchers performed multiple imputations to assess the outcome in patients with missing outcome data. The results indicated that the distribution of missing data in SNOT‐22 was balanced in both arms at 12 months (Data not shown).

**TABLE 2:** Baseline characteristics of the included studies.

	ESS (*n* = 234)	OSTRO (*n* = 410)	SINUS‐52 (*n* = 448)	SYNAPSE (*n* = 407)	POLYP‐OLE (*n* = 249)
Age (y)	50.5	50.1	52.0	48.7	50.8
Female (%)	39.3	35.9	37.7	35.0	35.7
Body‐mass index (kg/m^2^)	NA	27.9	27.9	28.2	NA
Duration of nasal polyps (y)	NA	NA	10.9	11.4	NA
Previous nasal surgery (%)*	68.4_a,b_	73.2_b_	58.3_a_	100.0_c_	59.0_a_
Time since previous nasal polyp surgery (y)*	NA	6.9_a_	8.2_b_	4.0_c_	NA
Prior SCS use in the past 12 months (%)*	98.3_a_	NA	64.5_b_	48.4_c_	22.1_d_
Comorbid asthma (%)*	56.0_a_	67.8_b, c, d_	59.6_a,d_	71.0_c_	57.0_a, b, d_
N‐ERD (%)	19.7	29.5	26.8	26.5	26.9
Atopic status (%)	53.8	54.3	NA	NA	NA
SNOT‐22 score (scale, 0–110)*	51.2_a_	69.2_b_	51.9_a_	64.0_c_	59.8_c_
Overall symptom score^a^	7.7	NA	8.0	9.0	NA
Nasal congestion severity^b^	7.3	2.6	2.4	8.9	2.4
Loss of smell severity^b^	8.6	NA	2.8	9.6	2.6
EQ‐5D‐5 L VAS, mm*	70.5	NA	63.9	NA	NA
Peak nasal inspiratory flow, L/min	93.0	NA	84.4	NA	NA
Lund–Mackay CT score (scale, 0–24)	18.4	NA	18.0	NA	NA
UPSIT score (scale, 0–40)	NA	NA	13.6	NA	13.2
Serum total IgE (IU/mL)	258.3	232.4	239.8	NA	174.3
Blood eosinophil count (10⁹/L)*	0.5_a_	0.4_b_	0.4_b_	0.4_b_	0.3_c_

*Note*: Data are mean or *n* (%). Overall symptom score was measured using a visual analog scale (VAS) of 0–100 and 0–10 with 5 symptoms (nasal obstruction, nasal discharge, throat mucus, and loss of smell) in ESS and SYNAPSE trials, and daily patient‐reported scores ranging from 0 to 12 of four symptoms (nasal congestion, loss of smell, and anterior/posterior rhinorrhea [each on a scale of 0–3]) in SINUS trial.

Abbreviations: CT, computed tomography; EQ‐5D‐5L, the 5‐level EuroQol five dimensions questionnaire; N‐ERD, NSAIDs Exacerbated Respiratory Disease; NA, not available; SCS, systemic corticosteroids; SNOT‐22, 22‐item Sino‐Nasal Outcome Test; UPSIT, University of Pennsylvania Smell Identification Test; VAS, visual analog scale.

^a^ The SINUS‐52, and ESS trials used a 0 to 10 VAS score; The POLYP‐OLE trial used a 0 to 12 TNSS score. We didn’t perform statistical analysis on this baseline characteristic.

^b^ The SINUS‐52, POLYP‐OLE, and OSTRO trials used a 0 to 3 categorical scale; The SYNAPSE and ESS trials used a 0 to 10 VAS score. We didn’t perform statistical analysis on these baseline characteristics as well.

*represents *p* value < 0.05. a, b, c, d, e The identical subscript letters represent a cluster of studies whose corresponding baseline characteristics does not differ significantly from each other at the 0.05 level.

**FIGURE 2:**
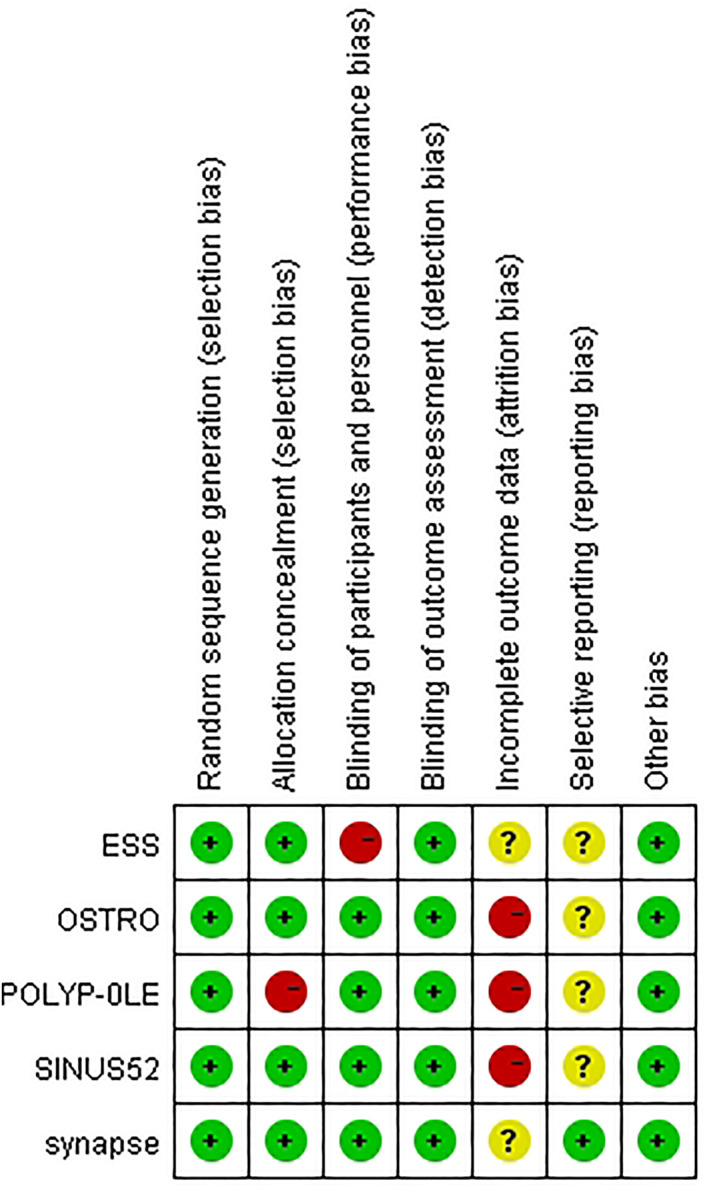
The risk of bias summary depicting the quality assessment of the included RCTs.

Overall, the risk of bias assessment showed that 5 included RCTs were good with a low risk of bias (shown in Figure 2 and eFigure 1). However, the attrition bias was not that optimistic, with three trials having high probabilities of discontinuation. Almost all the RCTs included in the meta‐analysis were phase 3 clinical trials and adopted a double‐blind research strategy, except for the study of ESS2022 in which no masking was applied owing to the nature of the study intervention.

### 3.2 ITC of primary and second outcome measures

#### 3.2.1 SNOT‐22

**FIGURE 3:**
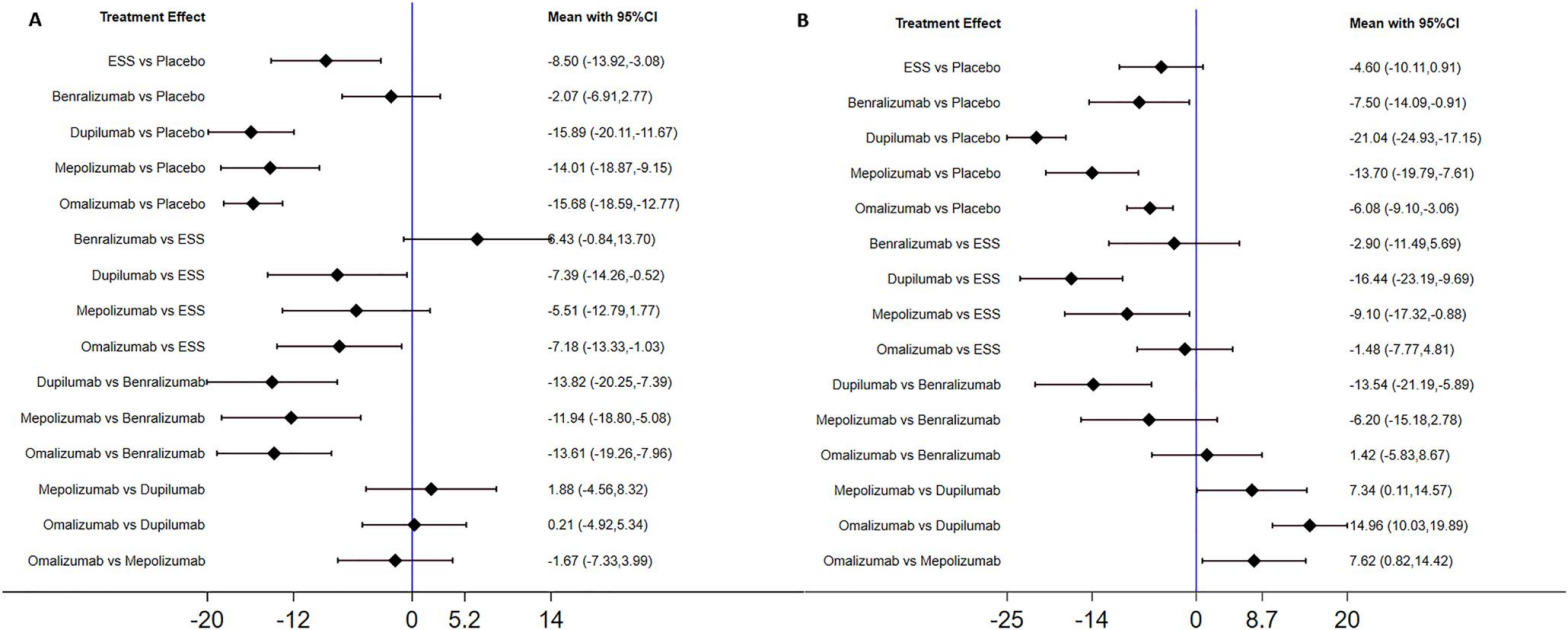
Indirect comparison of 22‐item sinonasal outcome test (SNOT‐22). Mean difference in SNOT‐22 from baseline to 6 months (A) and 1 year (B). CI, confidence interval; ESS, endoscopic sinus surgery.

SNOT‐22(scale 0–110), an indicator for evaluating patients' health‐related quality of life, was measured in all five trials at 6 months and 1 year. Quantitative analysis at two different time points showed significant improvements in almost all shared biological and surgical interventions except for Benralizumab at 6 months and endoscopic sinus surgery at 1 year (shown in Figure 3). The treatment effects at 6 months (MD [95% CI], −2.07 [−6.91, 2.77]) and 1 year (MD [95% CI], −7.50 [−14.09, −0.91]) for Benralizumab versus placebo indicated that the full benefit of intervention was not reached until after 6 months. Although causing less SNOT‐22 score reduction versus Dupilumab (MD [95% CI], 7.39 [0.52, 14.26] at 6 months and 16.44 [9.69, 23.19] at 1 year, ESS showed equivalent efficacy to other two biologics at 6 months (MD [95% CI], ESS vs. Mepolizumab, 5.51 [−1.77,12.79]; ESS vs. Benralizumab, −6.43 [−13.70,0.84]). Similarly, endoscopic sinus surgery did not present too much difference in reducing the SNOT22 score compared with Benralizumab and Omalizumab at 1 year.

#### 3.2.2 Symptom scores

**FIGURE 4:**
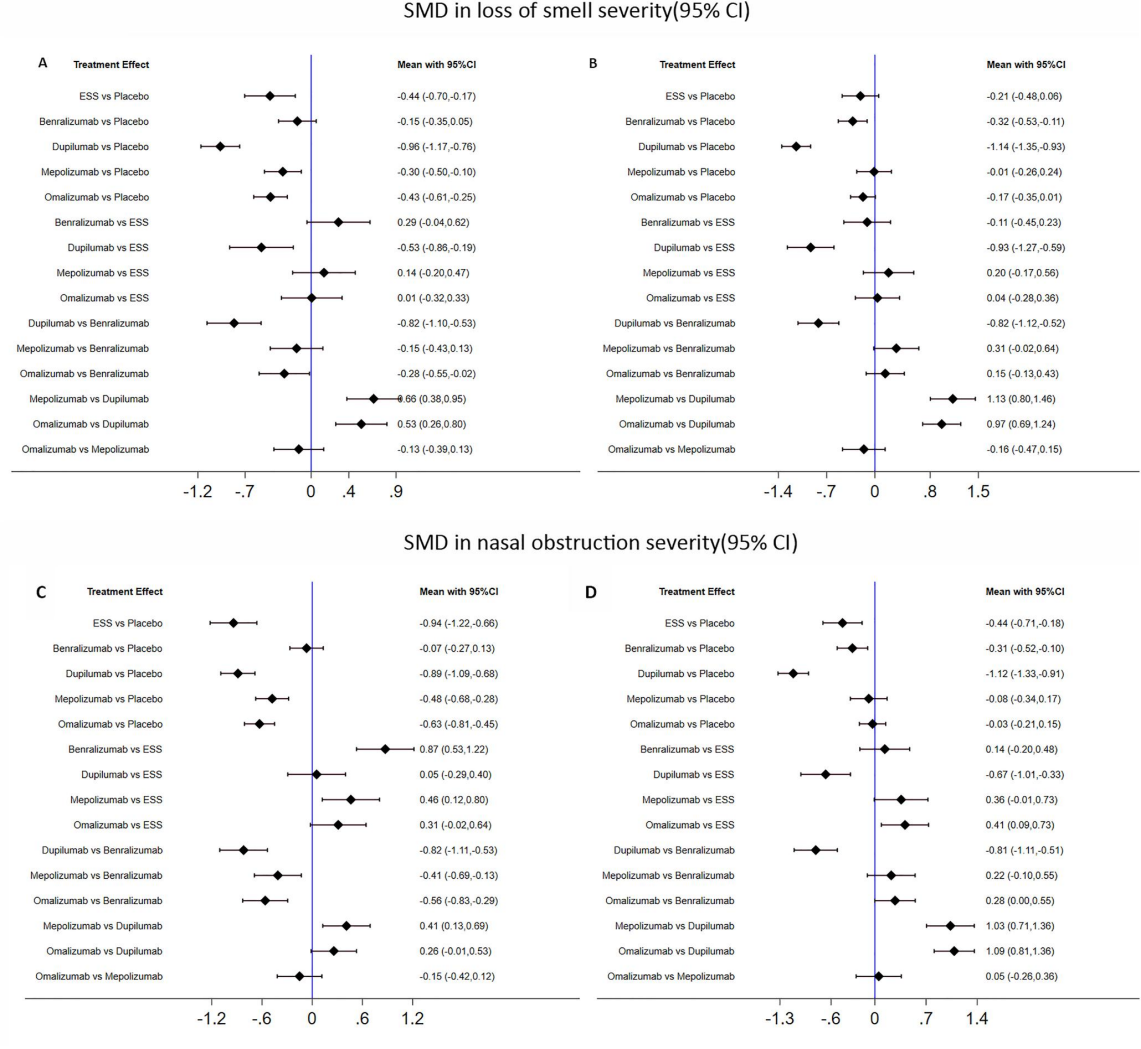
Indirect comparison of symptom severity in patients with CRSwNP: SMD in loss of smell severity from baseline to 6 months (A) and 1 year (B); SMD in nasal obstruction severity from baseline to 6 months (C) and 1 year (D). *Data measured using a visual analog scale (VAS) of 0–100 and 0–10 with 5 symptoms (nasal obstruction, nasal discharge, throat mucus, and loss of smell) in ESS and SYNAPSE trials, and daily patient‐reported scores ranging from 0 to 12 of four symptoms (nasal congestion, loss of smell, and anterior/posterior rhinorrhea [each on a scale of 0–3]) in both SINUS and POLYP trials. CI, confidence interval; ESS, endoscopic sinus surgery; SMD, standardized mean difference.

All 5 RCTs (*n* = 1748) evaluated the effect of ESS, Benralizumab, Dupilumab, Mepolizumab, and Omalizumab on symptom scores including loss of smell and nasal congestion as shown in Figure 4. For the loss of smell severity, biologics and ESS had an overwhelmingly positive effect to relieve the symptom versus placebo at 6 months except for Benralizumab, which did improve the loss of smell score at 1 year (SMD [95% CI], −0.32 [−0.53, −0.11]. Although a greater sense of smell improvement was observed in Dupilumab compared with ESS (SMD [95% CI], −0.53 [−0.86,−0.19] at 6 months and −0.93 [−1.27,−0.59] at 1 year), there were no significant differences between ESS and other biological cohorts at two different time points.

With regard to nasal congestion/obstruction scores, ESS, dupilumab, and omalizumab showed equivalent efficacy to improve the symptoms, while ESS resulted in statistically greater improvement in NCS than Benralizumab (SMD [95% CI], −0.87 [−1.22 to −0.53]) and Mepolizumab (SMD [95% CI], −0.46 [−0.80 to −0.12]) at 6 months. At EOF, nasal congestion/obstruction improvement favored ESS over Omalizumab and Mepolizumab (SMD [95% CI], −0.41 [−0.73 to −0.09] and −0.36 [−0.73 to 0.01], respectively), but was more likely to exist in Dupilumab compared with ESS.

#### 3.2.3 EQ‐5D‐5 L VAS score and PNIF

Three studies (*n* = 931 participants) assessed EQ‐5D‐5 L VAS score changes from baseline after interventions.^9^, ^11^, ^12^ As measured by EQ‐5D‐5 L VAS score, improvements in Dupilumab and Omalizumab compared with placebo were statistically significant at 6 months, but the superiority of Omalizumab over placebo vanished at 1 year. Additionally, Dupilumab and Omalizumab showed likely slight differences compared with ESS (MD [95% CI], 4.22 [−1.50–9.94], 2.90 [−2.51–8.31]) at 6 months.

**FIGURE 5:**
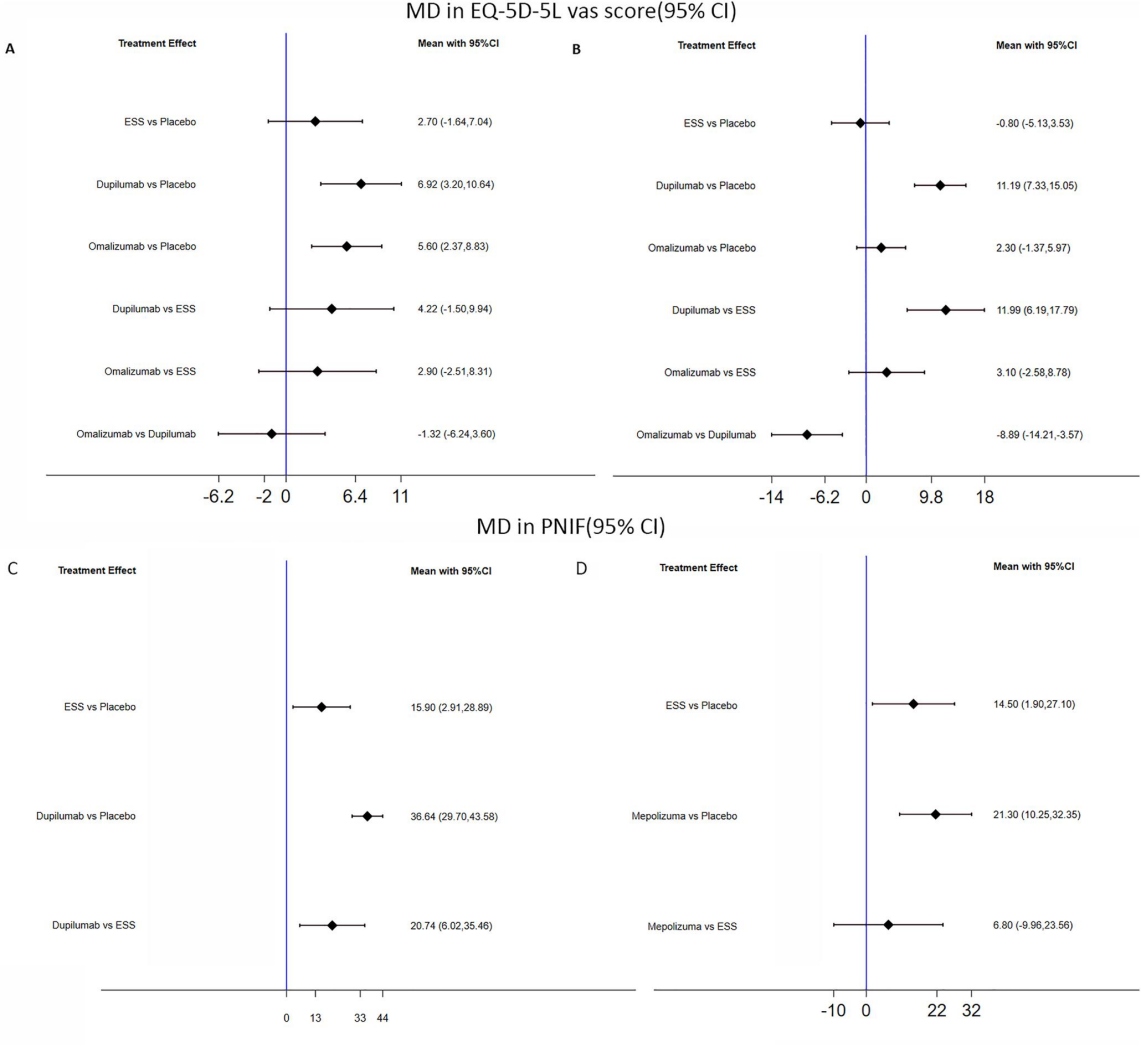
Indirect comparison of EQ‐5D‐5 L vas score and PNIF. Mean difference in EQ‐5D‐5 L vas score from baseline to 6 months (A) and 1 year (B); Mean difference in PNIF from baseline to 6 months (C) and 1 year (D) CI, confidence interval; ESS, endoscopic sinus surgery; EQ‐5D‐5 L, the 5‐level EuroQol five dimensions questionnaire; PNIF, peak nasal inspiratory flow.

Compared with placebo, evidence showed that ESS could improve the PNIF score both at 6 months and 1 year (MD [95% CI], 15.90 [2.91–28.89], and 14.50 [1.90–27.10], respectively) as Shown in Figure 5. But at 1 year, improvements in PNIF resulting from ESS and Mepolizumab were statistically similar.

#### 3.2.4 Probability of warranting remedial therapy

**FIGURE 6:**
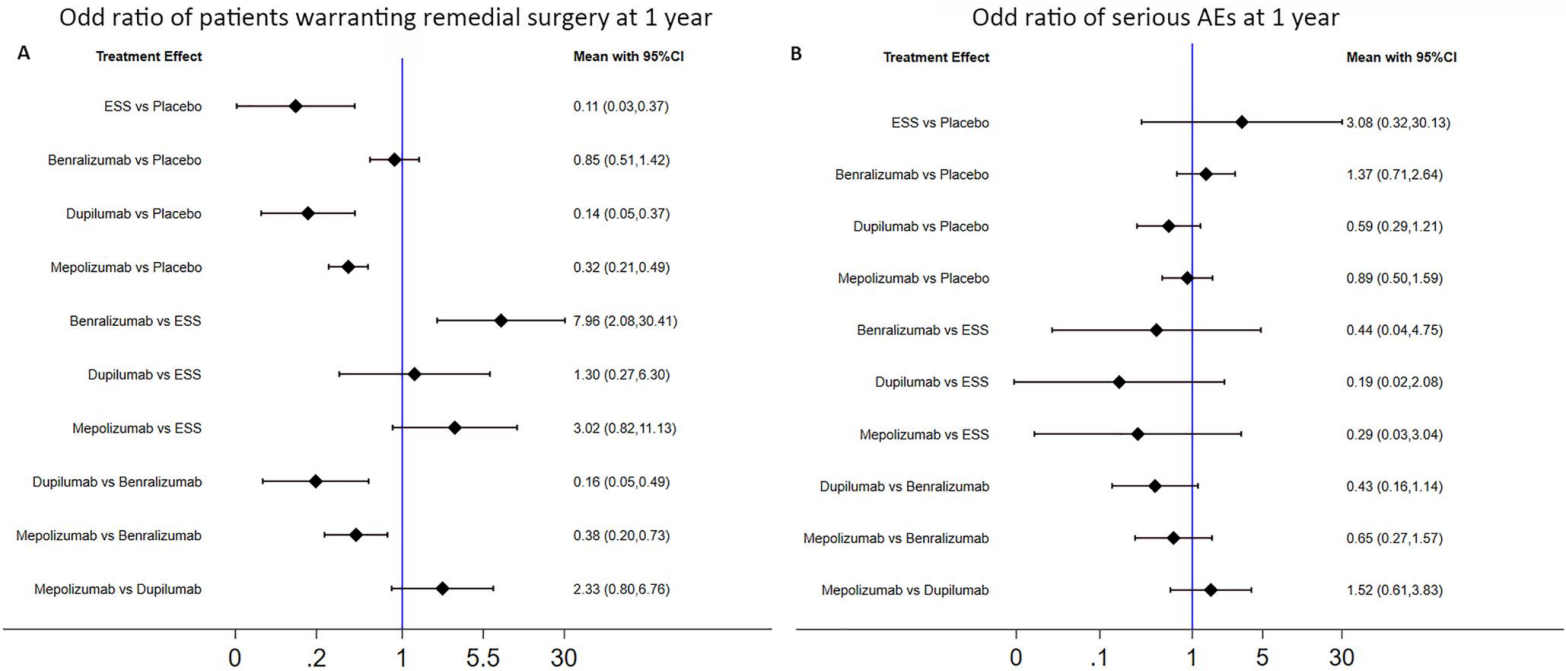
Indirect comparison of patients warranting remedial surgery and SAEs. Odd ratio of patients warranting remedial surgery (A) and SAEs (B) at 1 year. CI, confidence interval; ESS, endoscopic sinus surgery; SAEs, serious adverse events.

The probability of warranting remedial surgery was reported in ESS(12), Benralizumab,^10^ Dupilumab,^11^ and Mepolizumab^8^ trials, while the proportion of patients requiring rescue SCS and/or antibiotics was reported in all the five trials. Compared with placebo, ESS (OR [95%CI], 0.11 [0.03–0.37]), Dupilumab (OR [95%CI], 0.14 [0.05–0.37]) and Mepolizumab (OR [95%CI], 0.32 [0.21–0.49]) reduced remedial nasal polyps surgery. Based on the original data, the proportion of patients who warranted additional surgery was 2.9% with ESS versus 18.2% with Benralizumab (OR [95%CI], 0.13 [0.03–0.48]) at 1 year, although no significant difference was found between ESS and other biologics (Shown in Figure 6). Dupilumab was comparable to ESS therapy and ranked the best among mAbs for the reduced need of rescue SCS and/or antibiotics (shown in eFigure 3).

#### 3.2.5 Adverse events

**FIGURE 7:**
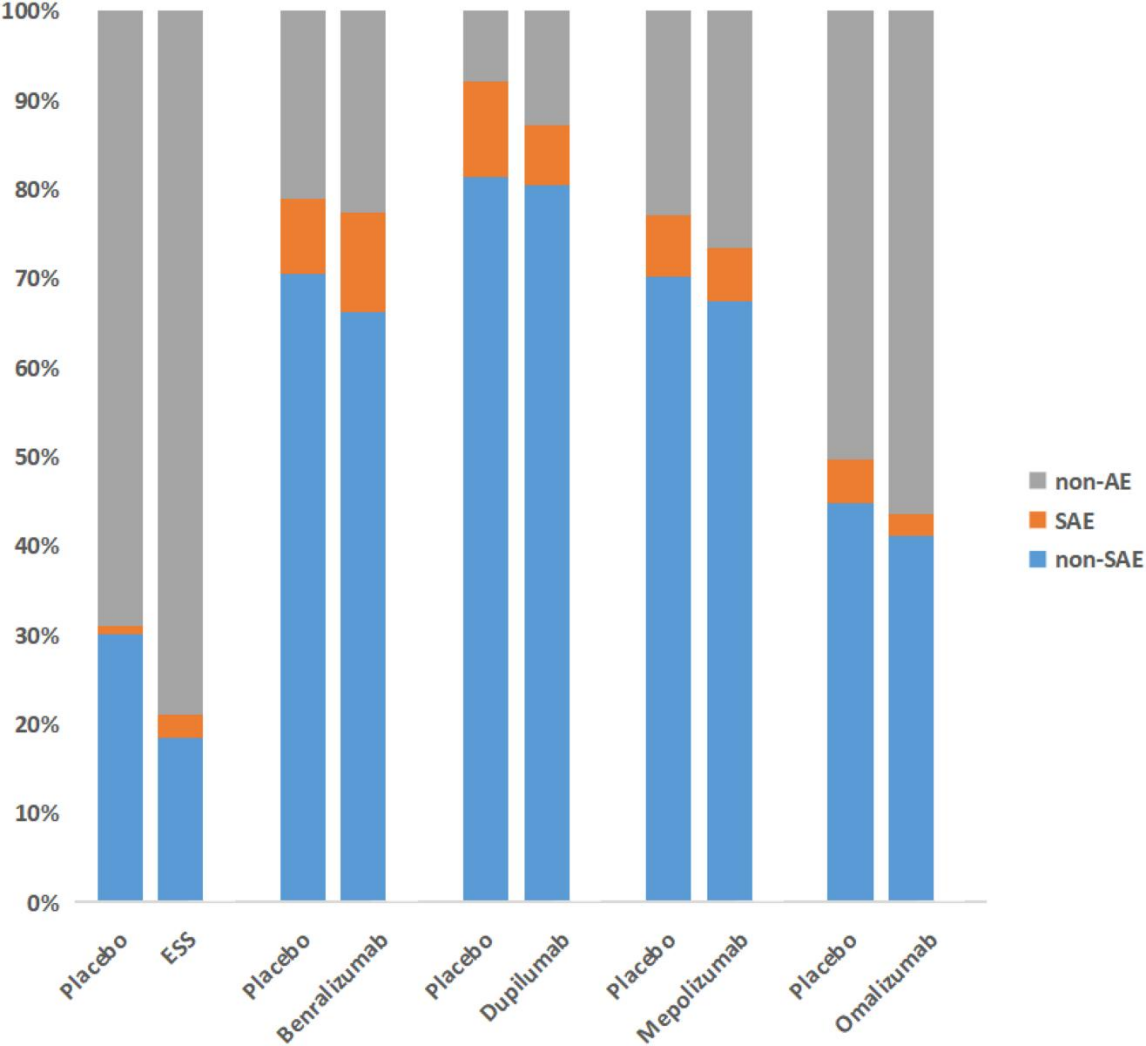
The percentage stacked bar chart of adverse events. The proportion of patients without adverse events (non‐AE) and patients who experienced serious adverse events (SAE) or other AEs except for SAEs (non‐SAE) among different groups.

We summarized the proportion of patients with or without AEs in Figure 7. Serious adverse event was usually known as adverse medical events at any dose that lead to death, are life‐threatening, require hospitalization or prolonged length of stay, result in persistent or severe disability/incapacity, are congenital anomalies, and are of medical significance. SAEs such as cardiac arrest, cerebrovascular accident, hand fracture, and so on were informed in all five trials. There was a similar incidence of both pooled and specific SAEs in each biologic and ESS, as illustrated in Figure 6 and Table 3. For the percentage of patients suffering from other (not including serious) AEs (shown in eFigure 4), there was no significant difference among all interventions.

**TABLE 3:** Adverse events.

	ESS (*n* = 109)	Benralizumab (*n* = 207)	Dupilumab(*n* = 295)	Mepolizumab (*n* = 206)	Omalizumab (*n* = 135)
SAEs					
Cardiac disorders	2 (2%)	3 (1%)	0	6 (3%)	0
digestive system diseases	0	8 (4%)	4 (1%)	3 (1%)	0
Injury, poisoning and procedural complications	0	0	5 (2%)	5 (2%)	2 (1%)
Infections and infestations	0	4 (2%)	6 (2%)	1 (0.5%)	0
asthma	0	1 (0.4%)	1 (0.3%)	0	1 (0.7%)
Blood and lymphatic system disorders	0	2 (1%)	1 (0.3%)	2 (1%)	0
Neoplasms benign, malignant and unspecified (incl cysts and polyps)	0	2 (1%)	1 (0.3%)	1 (0.5%)	0
Nervous system disorders	1 (1%)	3 (1%)	0	3 (1%)	0
Reproductive and urinary system disorders	0	2 (1%)	1 (0.3%)	3 (1%)	0
Eye disorders	0	1 (0.4%)	1 (0.3%)	0	0
Musculoskeletal and connective tissue disorders	0	2 (1%)	2 (0.7%)	0	0
Psychiatric disorders	0	1 (0.4%)	0	0	0
Epistaxis	0	1 (0.4%)	0	0	0
Eosinophilic granulomatosis with polyangiitis	0	0	1 (0.3%)	0	0
Pulmonary edema	0	0	0	1 (0.5%)	0
Type 2 diabetes mellitus	0	0	0	2 (1%)	0
Common AEs					
Headache	0	7 (3%)	31 (11%)	37 (18%)	11 (8%)
Nasopharyngitis		36 (17%)	64 (22%)	52 (25%)	8 (6%)
upper Respiratory tract infection		5 (2%)	18 (6%)	35 (17%)	8 (6%)
Other upper respiratory disorders	6 (6%)		68 (23%)	48 (23%)	7 (5%)
Asthma exacerbation/worsening		19 (9%)	23 (8%)	4 (2%)	5 (4%)
Injection site terms			34 (12%)	5 (2%)	7 (5%)
Musculoskeletal and connective tissue disorders			32 (11%)	28 (14%)	8 (6%)

*Note*: Common AEs were reported in either group while each SAE was enumerated clearly. Missing data indicates that the studies did not report the results, rather than the absence of such adverse events in corresponding groups.

Abbreviations: AE, adverse event; ESS, endoscopic sinus surgery; SAE, serious adverse event.

### 3.3 Sensitivity analysis in patients with a history of surgery

Since all the patients in the SYNAPSE trial had received prior surgery as required in the inclusion criteria, we performed a sensitivity analysis just to identify those results that were most susceptible to unsupported assumptions. In addition to Dupilumab and Mepolizumab in the SINUS‐52 and SYNAPSE trials, there were not feasible data reported for the comparison between other biologics. The results were in line with our findings originating from the whole population, indicating that Dupilumab had better efficacy than Mepolizumab at 6 months (SMD [95%CI], −0.30 [−0.54 to −0.05]) (shown in eFigure 2).

## 4 DISCUSSION

Although a consensus has been reached on the efficacy of ESS in treating CRSwNP, the unmet treatment need still exists due to a large proportion of patients presenting with uncontrolled disease postoperatively.^14^ The advent of biologics targeting type 2 (T2) inflammation has profoundly changed the treatment paradigm of CRSwNP. Although prior to our analysis, several meta‐analyses were performed to compare the efficacy and safety of different biologics for CRSwNP, comparisons, head‐to‐head trials in particular, between ESS and biologics were very limited.^14^, ^15^ In the current study, we firstly performed a network analysis to evaluate the outcomes between patients who underwent biological and surgical interventions using data from recently published RCTs.

There are several factors that may bring bias and make the meta‐analysis difficult. Firstly, the inherent property of ITC analysis could not fully reflect the real‐world clinical practice. Moreover, the heterogeneity of inclusion criteria and study populations in the RCTs may lead to the risk of bias. For example, the ESS RCT study offered surgical treatment to CRSwNP patients who had an indication for endoscopic sinus surgery judged by their ENT surgeon without a standardized criterion, while all the biologics RCT studies required total NPS ≥5. Thus, the percentage of more severe CRSwNP patients whose NPS ranged from 5 to 8 was 85% (198/233) and 100% in ESS and other biologic studies, which led to the imbalance of original baseline data. Although several heterogenous factors existed and possibly influenced our analysis, we found that the baseline features in the ESS group were comparable to those in biologic therapy in terms of previous nasal surgery, systemic corticosteroids usage, the percentage of comorbid asthma and Lund‐Mackay score. Additionally, the length of follow‐up was nearly equivalent and SNOT‐22 as the primary outcome in the meta‐analysis was available in all five studies. Therefore, these comparable baseline features and similar outcome measures encouraged us to perform the meta‐analysis.

In general, both biologics and ESS have demonstrated favorable outcomes compared with control therapies in improving key symptoms and QoL as measured by SNOT‐22, EQ‐5D, and VAS scores. Participants in the ESS trial had better or at least comparable Qol and symptoms benefit than most biologics except Dupilumab at two different time points. Safety data confirmed that both biologics and ESS are safe for the treatment of CRSwNP. Nevertheless, regarding some specific outcomes as presented in the results part, Dupilumab ranked among the most beneficial interventions compared with Omalizumab, Mepolizumab, and Benralizumab, while it had comparable efficacy in part of the key outcomes at specific time points.

NPS and NCS were the coprimary outcome measures in the phase 3 RCT trials of biologics, whereas the primary outcome was SNOT‐22 in the ESS cohort. The scoring system of nasal polyps in the endoscopic cohort is distinct from that in biologics studies, which makes the comparison of outcomes across trials inaccurate.^16^ Therefore, we did not include NPS as an efficacy endpoint in our analysis. A recent retrospective matched cohort study by Dharmarajan et al. showed that the ESS cohort is superior to Dupilumab treatment in the reduction of nasal polyp burden.^14^ However, a high proportion of patients would develop polyp recurrence and require revision surgery in the follow‐up period. In the current study, we chose changes from baseline in SNOT‐22 at the end of follow‐up as the primary endpoint. Recent meta‐analyses suggested that all biologic treatments could reduce the total SNOT‐22 score exceeding MCID. Dharmarajan et al. also found that the FESS cohort presented with an equivalent change of total SNOT‐22 score to patients treated with Dupilumab.^14^ A comparative analysis showed that ESS displayed significantly greater improvements in SNOT‐22 compared with dupilumab at 24 weeks, but without any significant difference at 52 weeks. However, our analysis suggested that Dupilumab had greater improvement in SNOT‐22 score versus ESS throughout one‐year follow‐up and demonstrated further improvement at 52 weeks compared to 24 weeks. This discrepancy may be caused by a higher baseline SNOT‐22 score in the two previous ESS cohorts, which may leave more room for improvement. Therefore, previous studies as well as our study demonstrated that dupilumab could offer at least comparable SNOT‐22 improvements in comparison with ESS. On the other hand, Dupilumab treatment showed more improvement in olfaction scores and extranasal rhinologic SNOT‐22 subdomain in comparison with the FESS cohort in a retrospective matched cohort study.^14^ Post‐hoc analysis of the SINUS‐24 and SINUS‐52 trials revealed that Dupilumab could also significantly improve sleep and functioning subdomains in CRSwNP patients.^17^, ^18^ The lack of subdomain results in ESS and biologic studies makes the comparison impossible for each subdomain in our analysis. Hence, further head‐to‐head trials or real‐world data are required to evaluate the efficacy of other SNOT‐22 domains between ESS and biologics.

The loss of smell and nasal congestion were the main symptoms significantly affecting patients' QoL.^17^ It is important to note in this network meta‐analysis that the positive impact of ESS and biologics upon cardinal nasal symptoms in CRSwNP was demonstrated. Miglani et al. demonstrated greater improvement of ESS than all the biologics including Dupilumab at 52 weeks, which was partly contrary to our results. The reason for the opposite outcomes between Dupilumab and ESS could not rule out the possibility of the different NCS scoring system in the ESS and biological groups.

As for olfactory function, Benralizumab significantly improved olfactory function for CRSwNP patients at 6 months, with the best efficacy for Dupilumab treatment. In contrast, the ESS, Benralizumab, and Omalizumab cohorts displayed limited efficacy to improve the debilitating symptoms in the long term. Three studies reporting mepolizumab, benralizumab, and omalizumab showed sustained benefits versus ESS in loss of smell management. Growing evidence suggests the role of type 2 inflammation in the pathogenesis of olfactory dysfunction in CRSwNP. ESS could efficiently eliminate polyps and open the paranasal sinuses, but not be able to reduce the inflammatory burden in the olfactory mucosa. However, biologics targeting type 2 inflammation have the advantage of relieving mucosal inflammation. In the current study, we could not compare the objective olfactory outcome because of the distinct measurement tools.

The EQ‐5D is a general quality of life measure which is sensitive to clinical change and has been adopted in CRS research.^19^ Our meta‐analysis revealed that Dupilumab treatment was superior to ESS as well as Omalizumab in the improvement of EQ‐5D VAS at the end of follow‐up. However, it should be noted that the EQ‐5D VAS scores at baseline in the ESS cohort were close to population norms (70.4–83.3) and higher than patients in the SINUS‐52 study, which left limited room for improvement.^20^ Rescue treatments such as oral corticosteroids or antibiotics and surgeries were reported in the ESS cohort, which allowed us to assess the exacerbation rate in these trials. We found a reduced need for rescue surgery for ESS, Dupilumab, and Mepolizumab in comparison with placebo treatment, but no significant difference existed among these treatments. The interpretation of this result should be cautioned due to the distinct definition of “need for surgery”. Similarly, ESS was equivalent to Dupilumab and Mepolizumab regarding the decreased necessity of rescue SCS and/or antibiotics.

Endoscopic sinus surgery for chronic rhinosinusitis has been developed for several decades and its safety has been confirmed. A recent meta‐analysis suggested that biologic treatment in CRSwNP reduced the risk of asthma exacerbations and had a similar incidence of adverse events to placebo groups, which indicated a great safety and tolerability profile of biologics.^21^ Although the spectrum of serious or non‐serious AEs was distinct between the ESS cohort and biologics, no significant difference for increased risk was observed in our pooled analysis. Since the follow‐up period in previous RCT studies was generally around 1‐year, further observations are still required to ascertain the long‐term safety of biologics in the application in CRSwNP.

Biologics face the greatest criticism of their cost and positioning in CRSwNP treatment strategies depending on cost‐effectiveness.^22^, ^23^ A real‐world study found that around one fifth of the asthmatics discontinued biological therapy in light of the heavy financial burden.^24^ Lacking cost‐effectiveness analysis, better efficacy of Dupilumab versus ESS in improving several nasal symptoms, and QoL may contribute to the abuse of biologics which used to be reserved only for ESS failure. A cohort‐style Markov decision‐tree economic model was implemented to compare the cost‐effectiveness in the treatment of CRSwNP, which reported that ESS was more cost‐effective than Dupilumab, with a cost of $50,436 for 9.80 quality‐adjusted life year (QALY) versus $536,420 for 8.95 QALYs.^25^ Maybe those who underwent biological therapy spent around 10 times the cost or $1870 more annually on CRSwNP and probably only got similar or even worse efficacy in QALYs compared with ESS.^26^ Similarly, Dupilumab induced an €21,817/QALY of incremental cost‐utility ratio compared to surgery plus conventional adjuvant therapy.^27^ In aggregate, biologics like Dupilumab are clinically effective alternatives rather than cost‐effective strategies. Although a de‐escalation strategy with an increased dosing interval could reduce the direct cost and decrease the financial burden,^28^ unfortunately, biological drugs are still not a “curative” treatment therapy for CRSwNP, which indicates that continuous biological treatment is still warranted for the maintenance of therapeutic benefits for this disease. Future studies are imperative to investigate both direct and indirect costs between ESS and biologics. In real‐world clinical practice, the benefits, risks, and costs should be integrated into the patient‐centered decision‐making process in choosing appropriate treatment modalities.

Given the trend toward incorporating biologics into health care delivery over the coming years, it is imperative to define the indication of biologics in CRSwNP management. According to the document recently proposed by the European Forum for Research and Education in Allergy and Airway Diseases (EUFOREA),^29^ biologics are an effective alternative treatment modality for patients with uncontrolled severe T2 CRSwNP. Our study demonstrated the potential value of biologics compared with ESS in such patients after failure of first‐line treatment including medical therapy and (or) ESS. However, the utility of these biologics is endotype‐targeted and is now only approved in patients with T2 CRSwNP. Although inflammatory pathways are not mutually exclusive,^30^ such as the interaction between eosinophilic and neutrophilic inflammation, whether the therapeutic effects of these T2 biologics in non‐type 2 CRSwNP remains an area of active investigation. Collectively, biological agents are more suitable as a post‐failure countermeasure after traditional treatment for managing T2 CRSwNP. Meanwhile, due to the heterogeneity of immunopathogenesis of CRSwNP, a one‐size‐fits‐all solution would have constituted a major departure from the principles of precision medicine. The prescription of biologics to CRSwNP patients also depends greatly on local circumstances such as funding, medical insurance, regulations, and availability of biologics.

Although we screened out high‐quality RCTs and applied validated ITC analysis in the current study, several limitations still need to be clarified. The first issue was that subgroup data such as asthma status was unavailable in the ESS cohort, which precluded the sensitive analysis and subgroup analysis. A comparison between VAS scales using continuous data and semi‐quantitative Likert scales using ordinal data for many outcomes such as nasal obstruction and loss of smell severity limits the ability to reach valid conclusions. Finally, the follow‐up period was no more than 1 year. The long‐term efficacy and safety of biologics in the treatment of CRSwNP are required to be evaluated.

## 5 CONCLUSION

In conclusion, our meta‐analysis suggested that both ESS and biologics presented great efficacy to improve key symptoms and quality of life in patients with CRSwNP for around 1‐year treatment period. Our findings provided new evidence that biologic therapy, especially Dupilumab, could be taken into consideration for selected patients with recurrent and uncontrolled disease after first‐line treatment. More head‐to‐head or real‐world studies are required to evaluate the application scenarios of biologics such as combinational treatment and compare the efficacy as well as cost‐effectiveness with surgical treatment in different endotypes of CRSwNP patients.

## AUTHOR CONTRIBUTIONS

Huan Wang, Li Hu, Hongmeng Yu and Xicai Sun contributed to the conception of the study; Jiani Chen and Huan Wang contributed significantly to analysis and manuscript preparation; Jiani Chen, Le Shi and Chen Zhang contributed significantly to implement the computer code and testing of existing code components; Jiani Chen and Qianqian Zhang contributed significantly to the visualization and data presentation; Jiani Chen, Huan Wang, Xiaole Song and Dehui Wang helped perform the analysis with constructive discussions.

## CONFLICT OF INTEREST STATEMENT

The Authors declare no conflicts of interest.

6

## SYSTEMATIC REVIEW REGISTRATION

This study is registered with the PROSPERO, CRD42022365943.

## Supporting information

Supporting Information S1

Supporting Information S2

Supporting Information S3

Supporting Information S4

Supporting Information S5

## Data Availability

The data that support the findings of this study are openly available in Elsevier at http://doi.org/10.1016/S2213‐2600(21)00097‐7, http://doi.org/10.1016/j.jaci.2021.07.045, http://doi.org/10.1016/j.jaci.2021.08.030, http://doi.org/10.1016/S0140‐6736(19)31881‐1, http://doi.org/10.1016/S2213‐2600(21)00457‐4, reference number 7‐11, respectively. Further enquiries can be directed to the corresponding author.
