# Supplementary material for: Comparative short‐term efficacy of endoscopic sinus surgery and biological therapies in chronic rhinosinusitis with nasal polyps: A network meta‐analysis
Source: Clin Transl Allergy. 2023 Jun 1;13(6):e12269. doi: 10.1002/clt2.12269 (PMC10234113; doi:10.1002/clt2.12269)
Supplement: Supplementary file 1 — Supporting Information S1 [file CLT2-13-e12269-s005.docx]

CI: confidence interval

CRG: control group

CRSwNP: Chronic rhinosinusitis with nasal polyps

CT: computed tomography

ENT: ears, nose and throat

EOF: end of follow-up

EQ-5D-5L: the 5-level EuroQol five dimensions questionnaire

ESS: endoscopic sinus surgery

EUFOREA: the European Forum for Research and Education in Allergy and Airway Diseases

HRQoL: Health-related Quality of Life

IgE: immunoglobulin E

INCS: intranasal corticosteroid

ITC: indirect treatment comparison

MD: mean difference

MFNS: Mometasone furoate nasal spray

NA: not available

NCS: Nasal Congestion Score

N-ERD: NSAIDs Exacerbated Respiratory Disease

NPS: nasal polyp score

OR: odd ratio

PNIF: peak nasal inspiratory flow

Q2W: every 2 weeks

Q4W: every 4 weeks

QALY: quality-adjusted life year

QoL: quality of life

RCT: randomized controlled trial

SAE: serious adverse event

SC: subcutaneous

SCS: systemic corticosteroids

SD: standard deviation

SMD: standardized mean difference

SNOT-22: the 22-item Sino-Nasal Outcome Test

T2: type 2

TRG: treatment group

UPSIT: University of Pennsylvania Smell Identification Test

VAS: visual analogue scale
