# Supplementary figures and images for: Comparative short‐term efficacy of endoscopic sinus surgery and biological therapies in chronic rhinosinusitis with nasal polyps: A network meta‐analysis
Source: Clin Transl Allergy. 2023 Jun 1;13(6):e12269. doi: 10.1002/clt2.12269 (PMC10234113; doi:10.1002/clt2.12269)

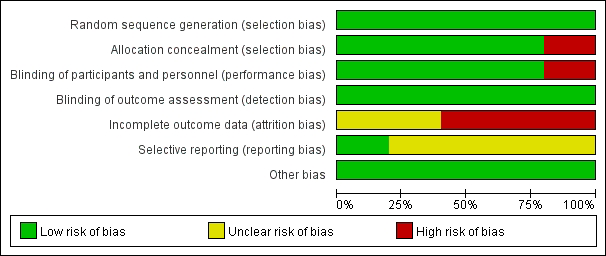

Supplement: Supplementary file 2 — Supporting Information S2 [file CLT2-13-e12269-s003.tif]

# Treatment Effect

# Mean with 95%CI

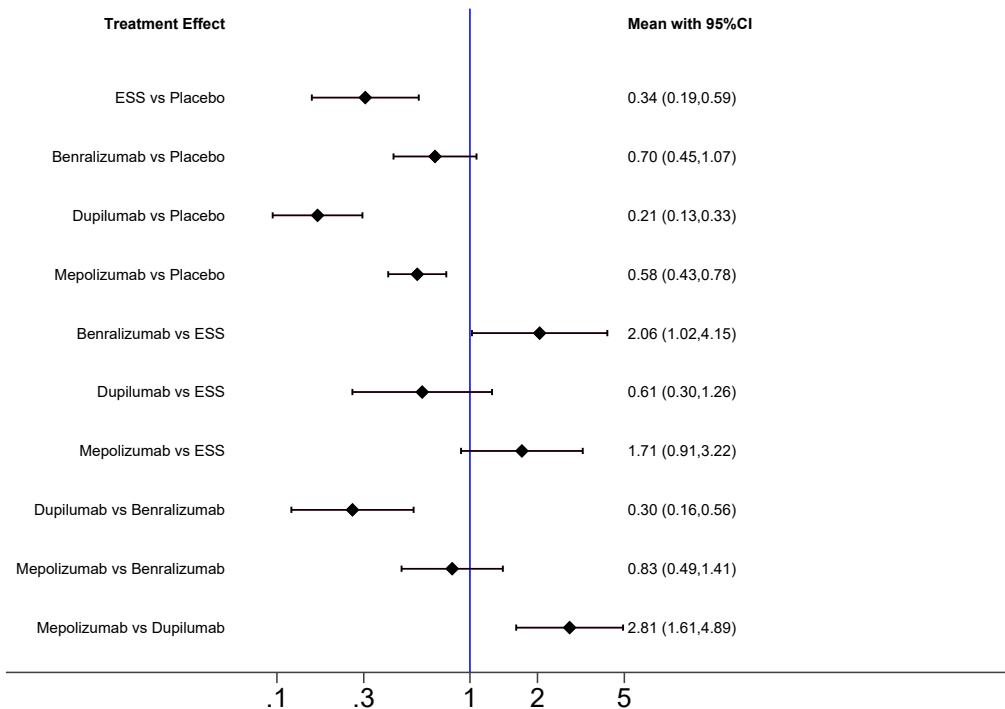

Supplement: Supplementary file 4 — Supporting Information S4 [file CLT2-13-e12269-s001.pdf]

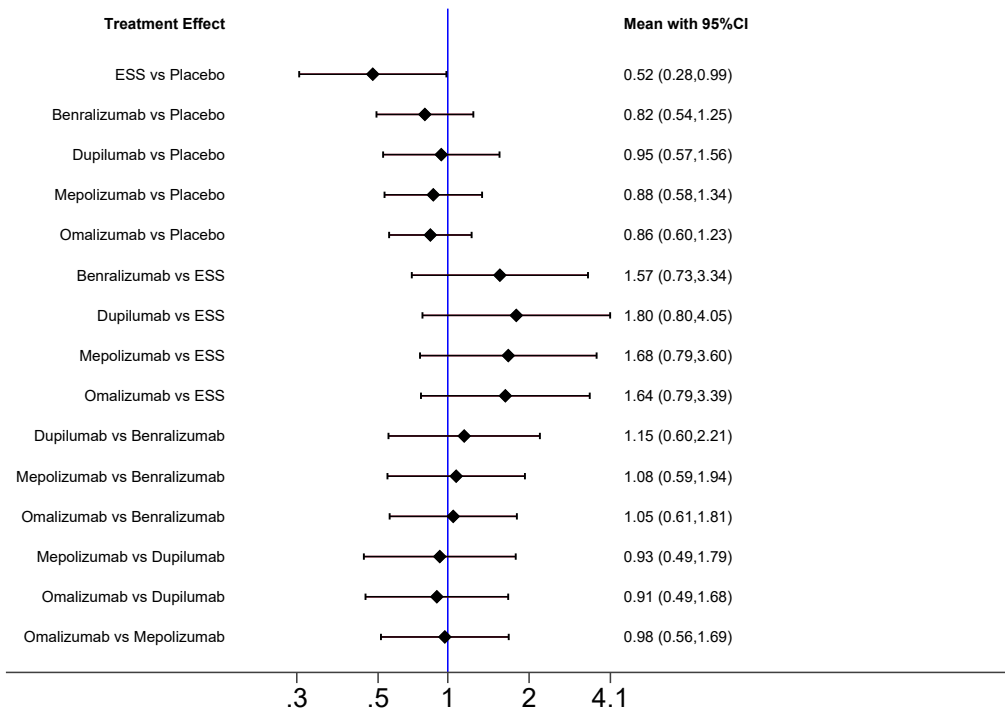

Supplement: Supplementary file 5 — Supporting Information S5 [file CLT2-13-e12269-s004.pdf]
