# Supplementary material for: Comparative short‐term efficacy of endoscopic sinus surgery and biological therapies in chronic rhinosinusitis with nasal polyps: A network meta‐analysis
Source: Clin Transl Allergy. 2023 Jun 1;13(6):e12269. doi: 10.1002/clt2.12269 (PMC10234113; doi:10.1002/clt2.12269)
Supplement: Supplementary file 3 — Supporting Information S3 [file CLT2-13-e12269-s002.pdf]

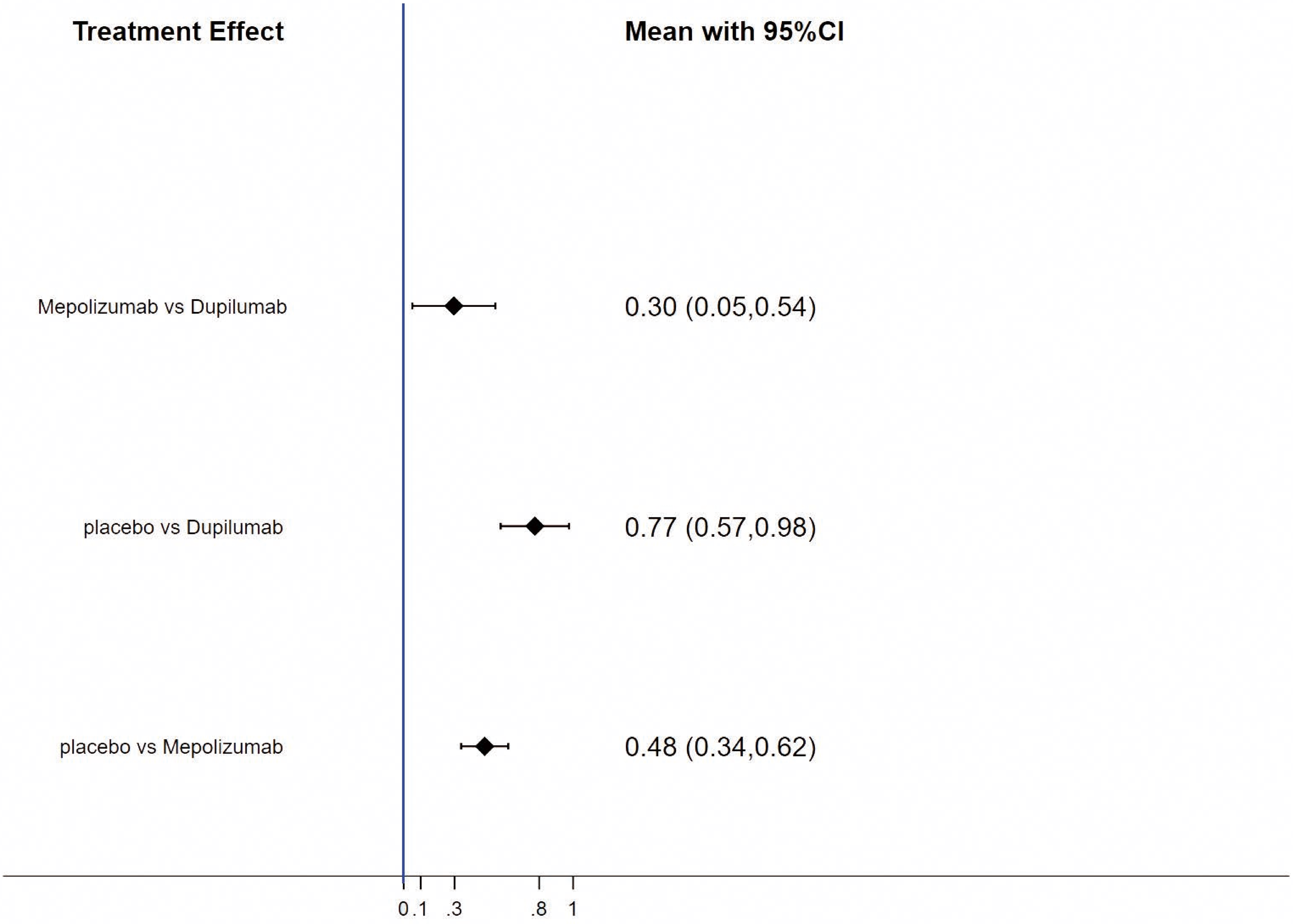
Treatment Effect
Mean with 95%CI
Mepolizumab vs Dupilumab
0.30 (0.05,0.54)
placebo vs Dupilumab
0.77 (0.57,0.98)
placebo vs Mepolizumab
0.48 (0.34,0.62)
0
.1
.3
.8
1
